# Hepatitis B Infection, Eastern India

**DOI:** 10.3201/eid1007.030766

**Published:** 2004-07

**Authors:** Kamalesh Sarkar, Dwijendra N. Ganguly, Baisali Bal, Malay K. Saha, Sujit K. Bhattacharya

**Affiliations:** * National Institute of Cholera and Enteric Diseases, Kolkata, West Bengal, India;; † Kolkata National Medical College, Kolkata, West Bengal, India

**Keywords:** letter, HBV, HIV, commercial sex workers, brothel, vaccine

**To the Editor:** The National Institute of Cholera and Enteric Diseases (ICMR), Kolkata, India, conducted a serologic study in July 2003 to determine the rate of hepatitis B virus (HBV) infection of brothel-based commercial sex workers. These study participants worked in the South-24 Parganas district of West Bengal, one of the eastern states of India. Routine immunization to prevent HBV infection is not a practice in India, and chronic HBV infection is endemic (1). The nature of their work makes commercial sex workers more vulnerable to HBV infection, which could accelerate the infection’s spread into the general community, particularly in areas with low literacy rates and socioeconomic status.

The study participants were 167 commercial sex workers from three prominent brothels, which were located in small towns and along the national highway of the district. Blood samples from the participants were tested by using the HBsAg unlinked anonymous method. The results showed that 23.3% (unpub. data, National Institute of Cholera and Enteric Diseases) of the commercial sex workers were infected with HBV. The Figure shows the distribution of commercial sex workers and prevalence of HBV infection by age group.

**Figure:**
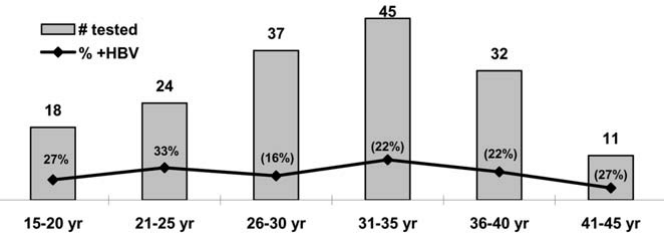
Distribution of sex workers by age and HBV infection (n = 167)

To ascertain a possible route of transmission other than sexual activity, all study participants were questioned about their medical history, including jaundice, injury requiring blood transfusion, surgical events, and tattooing. The evidence indicated that HBV infection in the study participants was not acquired by any route other than sexual activity. Since the carrier rate of chronic HBV in the general population is approximately 5% in eastern India (2), this increase in chronic HBV infection can be attributed to sexual transmission.

The Figure indicates that HBV infection is not correlated to age or duration of commercial sex work; the rate of infection is distributed almost equally in all age groups except the 26- to 30-year-old group in which it was slightly lower. Most of the HBV-infected commercial sex workers likely were infected early in their profession and remained infected throughout their lives, which led to higher rate of chronic infection as compared with the general population.

This higher rate of chronic HBV infection among commercial sex workers (23.3%) is of concern, particularly in a country with an estimated 4.58 million persons infected with HIV. In India, >80% of HIV infection is transmitted sexually (3). HIV infection can affect the natural course of HBV; sexual activity between HIV- and HBV-infected persons could prolong the infected status of those infected with HBV, as was shown in a previous study (4). This sexual activity could facilitate HBV transmission, particularly in areas that have few resources or where the rate of condom use is low or questionable.

After this study concluded, all study participants were notified of their infection status and advised to use condoms when engaging in sexual activity. The commercial sex workers and their family members were advised that they should be tested for HBV infection and receive HBV vaccination if test results were negative. Local health authorities were advised that commercial sex workers and their clients should be vaccinated to prevent HBV infection.
